# ISGylation in Innate Antiviral Immunity and Pathogen Defense Responses: A Review

**DOI:** 10.3389/fcell.2021.788410

**Published:** 2021-11-25

**Authors:** Mengdi Zhang, Jingxian Li, Haiyan Yan, Jun Huang, Fangwei Wang, Ting Liu, Linghui Zeng, Fangfang Zhou

**Affiliations:** ^1^ School of Medicine, Zhejiang University City College, Hangzhou, China; ^2^ MOE Laboratory of Biosystems Homeostasis and Protection and Innovation Center for Cell Signaling Network, Life Sciences Institute, Zhejiang University, Hangzhou, China; ^3^ Institute of Biology and Medical Sciences, Soochow University, Suzhou, China

**Keywords:** ISG15, isgylation, immune response, innate antiviral immunity, SARS PLpro

## Abstract

The interferon-stimulating gene 15 (ISG15) protein is a ubiquitin-like protein induced by interferons or pathogens. ISG15 can exist in free form or covalently bind to the target protein through an enzymatic cascade reaction, which is called ISGylation. ISGylation has been found to play an important role in the innate immune responses induced by type I interferon, and is, thus, critical for the defense of host cells against RNA, DNA, and retroviruses. Through covalent binding with the host and viral target proteins, ISG15 inhibits the release of viral particles, hinder viral replication, and regulates the incubation period of viruses, thereby exerting strong antiviral effects. The SARS-CoV-2 papain-like protease, a virus-encoded deubiquitinating enzyme, has demonstrated activity on both ubiquitin and ISG15 chain conjugations, thus playing a suppressive role against the host antiviral innate immune response. Here we review the recent research progress in understanding ISG15-type ubiquitin-like modifications, with an emphasis on the underlying molecular mechanisms. We provide comprehensive references for further studies on the role of ISG15 in antiviral immunity, which may enable development of new antiviral drugs.

## Introduction

Interferon-stimulated gene 15 (ISG15) is a member of the family of interferon-stimulating genes (ISGs) (Takeuchi et al., 2019), which are fast and strong type I interferon (IFN)-stimulated reaction proteins that inhibit viral replication, whose function against virus invasion has been fully investigated (Loeb and Haas, 1992; Hermann and Bogunovic, 2017; Sooryanarain et al., 2017). Viral infection induces IFN synthesis, and the secreted IFN acts on nearby uninfected cells to resist the infection. After the virus enters the body, IFN binds to IFN receptors, which activate the Janus protein tyrosine kinase-signal transducer and activator of transcription pathway to form the interferon-stimulating factor 3 complex, which induces the expression of hundreds of ISGs, including ISG15, which can fight against the replication and invasion of the virus (Yuan and Krug, 2001).

**FIGURE 1:**
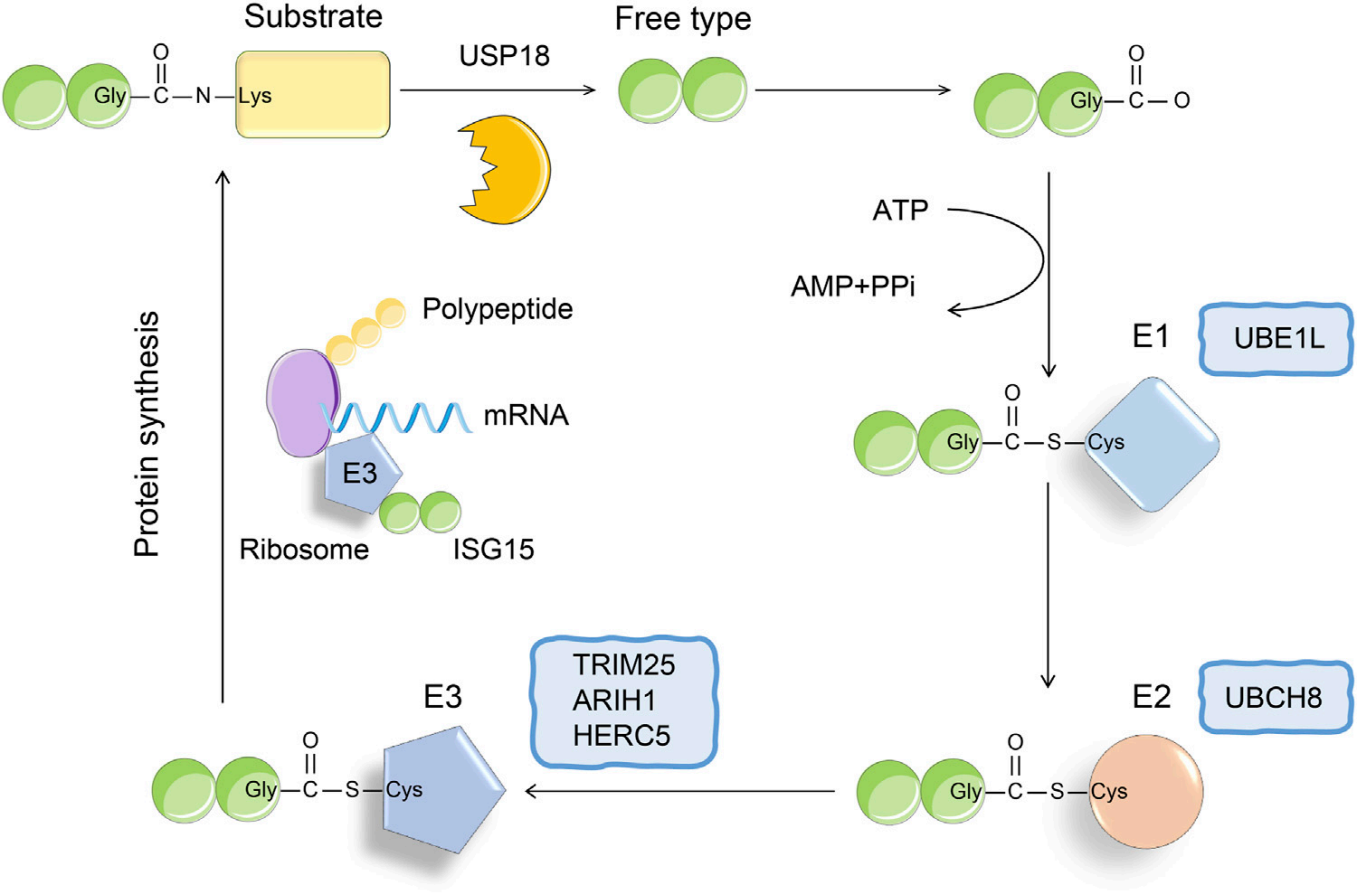
The conjugation of ISG15. The binding process of ISG15 and substrate is similar to the three-step enzyme cascade reaction of ubiquitination. The formation of thioester bond between E1 activating enzyme (UBE1L) and ISG15 depends on ATP, thereby activating ISG15. Next, SIG15 is transferred to the cysteine active site of E2 ligase (UBCH8). Finally, E3 ligase binds to polysomes, thereby promoting the binding of ISG15 to the nascent target protein. The process of ISGylation is reversible, and USP18 as a deubiquitinating enzyme can specifically remove ISG15 from the binding protein.

Recently, the function of ISG15 as a ubiquitin-like protein has attracted much attention. ISG15 is the first identified ubiquitin-like protein, which contains two ubiquitin-like domains, and its amino acid sequence shows 50% homology with ubiquitin (Dos Santos and Mansur, 2017). Under physiological conditions, the ISG15 precursor protein can be cleaved into a mature 15-kDa form, exposing the carboxyl-terminated LRLRGG motif, which recognizes and binds to substrate lysine residues, resulting in its ISGylation (Figure 1) (Potter et al., 1999; Langevin et al., 2013; Zuo et al., 2016). Similar to ubiquitin modification, ISG modification of the substrate is also catalyzed by ubiquitin-activating enzyme E1, ubiquitin-binding enzyme E2, and ubiquitin ligase E3 (Mustachio et al., 2018). The removal of substrate ISGylation is catalyzed by deubiquitinases (DUB), and ubiquitin-specific peptidase 18 (USP18; also called UBP43) is a human-specific enzyme that removes ISG15 from conjugated proteins (Malakhov et al., 2002; Basters et al., 2017; Basters et al., 2018; Mustachio et al., 2018). Using ISG15 as bait, we obtained more than 300 candidate ISG15 substrates using immunoprecipitation-mass spectrometry. At present, more than 100 proteins have been established as substrates of ISG15, including p53, nuclear factor kappa B (NF-κΒ), KRAS, cyclin D, PTEN protein, STAT1, and retinoic acid-induced gene I (RIG-I) (Feng et al., 2008; Kim et al., 2008; Huang et al., 2014; Ganesan et al., 2016; Park et al., 2016; Mustachio et al., 2018). In this review, we discuss how ISG15 regulates viral replication, inflammation, cell proliferation and differentiation, and tumor genesis and development by modifying these proteins.

Currently, there are still many controversies regarding whether ISG15 exerts a tumor-suppressing effect or a cancer-promoting effect. Both unconjugated and conjugated ISG15 have demonstrated tumor-suppressing and cancer-promoting functions. Research results show that the tumor-suppressing function of unconjugated ISG15 is mainly related to its immune regulatory function (Desai, 2015). Yeung TL using laser microdissection and sequencing analysis that free ISG15 was highly expressed in serous ovarian cancer with high infiltration of CD8^+^ T cells (Yeung et al., 2018). *In vitro* experiments indicated that free ISG15 can increase the ISG modification of extracellular signal-regulated protein kinase one and the viability of natural killer (NK) cells and CD8^+^ T cells and enhance immune surveillance (Burks et al., 2015). Moreover, studies have shown that unconjugated ISG15 exerts a cancer-promoting function by enhancing the stem transformation and proliferation of tumor cells (Sainz et al., 2014; Chen et al., 2016). The same effect occurs in conjugated ISG15, which exerts a cancer-promoting effect by interacting with carcinogens (Burks et al., 2014), and a tumor-suppressing effect by regulating the function of p53 (Park et al., 2016; Jeon et al., 2017). Therefore, ISG15 can perform distinct functions depending on the cell type and physiological state, substrate, and subcellular location.

## ISG15 and Innate Immunity

**FIGURE 2:**
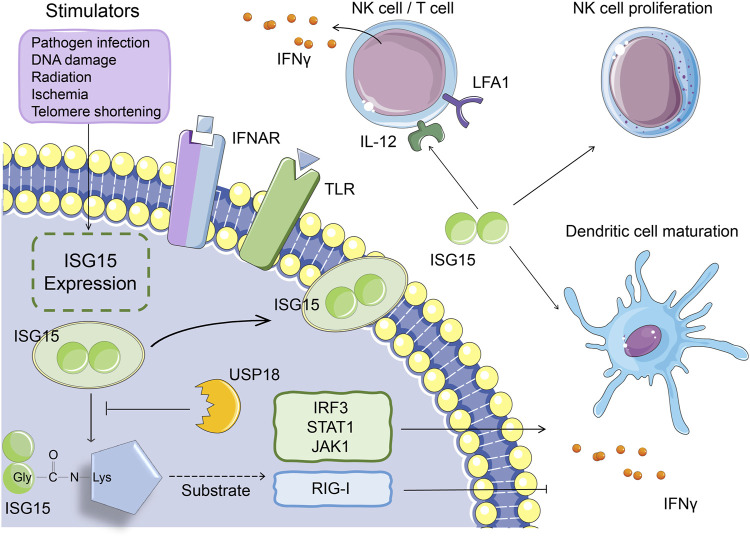
The function of ISG15 in immune response. Under pathogenic stimuli such as viral and bacterial infections, LPS, and DNA damage, monocytes, lymphocytes, neutrophils, etc. can all secrete ISG15. Intracellular ISG15 can bind to proteins related to innate immune signaling pathways, activate IRF3, STAT1, JAK1 and other proteins, or inhibit protein activity (such as RIG-I), thereby promoting or inhibiting the secretion of IFNγ. The ISG15 secreted *in vitro* can bind to the LFA1 receptor on the cell surface, thereby promoting the secretion of IFNγ from NK cells and T cells. It can also induce the proliferation of NK cells and the maturation of dendritic cells.

Studies have shown that fibroblasts, monocytes, lymphocytes, neutrophils, plasma cells, and NK cells secrete small amounts of ISG15 under physiological conditions (Bogunovic et al., 2012). In addition, the expression of ISG15 can be affected by many factors. Viral and bacterial infections, LPS, DNA damage and other pathogenic stimuli can activate the expression of ISG15 (Malakhova et al., 2002; Pitha-Rowe et al., 2004). The free form of ISG15 binds to the LFA1 receptor on the surface of NK cells and T lymphocytes, increasing the release of type I and II IFNs and activating natural and acquired immunity (Swaim et al., 2017). ISG15 can also induce the proliferation of NK cells, the IFNγ production of NK cells and T cells, the maturation of dendritic cells, and enhancement of antigen presentation and function as a chemokine that promotes the enrichment of neutrophils to inflammatory regions (Figure 2) (Morales et al., 2015; D'Cunha et al., 1996; Padovan et al., 2002; Owhashi et al., 2003; Recht et al., 1991).

Proteomic studies have identified that the immune-regulating factors interferon-regulated transcription factor 3 (IRF3), STAT1, and Janus kinase one function as substrates of ISG15 and that the ISGylation of these proteins increases the release of type I IFNs and ISGs, thereby extending the immune response signal cascade (Ganesan et al., 2016; Albert et al., 2018; Yoo et al., 2018; Malakhov et al., 2003). For example, when the host is infected, STAT1 ISGylation promotes the maintenance of phosphorylation and continuous activation of downstream signaling, which ultimately promotes a more powerful IFN response (Ganesan et al., 2016). In addition to positive regulation, ISG15 negatively regulates type I IFN signaling at multiple levels, such as ISGylation of the RIG-I protein, which inhibits IFN expression (Figure 2) (Zhao et al., 2005; Kim et al., 2008; Zhu et al., 2014; Du et al., 2018). On the one hand, because the process of covalent binding of ISG15 to the target protein is reversible, this binding can be dissociated by the ubiquitin-specific protease USP18, which indirectly regulates IFN expression. On the other hand, the deubiquitinating enzyme USP18 can also directly inhibit type I IFN receptor signaling, thereby suppressing the immune response (Arimoto et al., 2017). The non-covalent interactions of ISG15 and USP18 prevent the ubiquitination of USP18 by S-phase kinase-associated protein two and stabilize the downregulation of the IFN signaling pathway by USP18 (Tokarz et al., 2004; Zhang et al., 2015).

These results suggest that ISG15 can regulate immune function from multiple perspectives, such as stimulating immune cell maturation, regulating cytokine release, and affecting IFN signaling. In recent years, many studies have explored the role of ISG15 in antiviral innate immunity, especially in the process of viral infection, and the role of ISGylation of host and viral target proteins in immune defense. In this review, we explore this topic in detail.

## Antiviral Effects of ISGylation on Host Proteins and Their Functions

Although the ISG15 protein was discovered in 1979, its nature and function were not elucidated for many years, until researchers discovered that IFN-induced ISG15 and its covalent form were implicated as a central player in the process of viral infection. Gene knockout, overexpression, genetic deletion of each component in the ISG15 cascade reaction process, and various other methods have since been used to determine whether ISG15 is involved in the host antiviral immune response (Campbell and Lenschow, 2013).

ISG15 can affect the antiviral immune response by binding to the target proteins of the IFN, NF-κB, and c-Jun N-terminal kinase (JNK) pathways (Jeon et al., 2009). Among them, the key factor for type I IFN response, IRF3, is a target of ISG15. The combination of ISG15 and IRF3 inhibited the proteasomal degradation of IRF3 and enhanced the intracellular IFN response (Ganesan et al., 2016). Concurrently, the covalent binding of ISG to the antiviral effector molecules K193, K360, and K366 can weaken the interaction between IRF3 and peptidyl-prolyl-cis-trans isomerase one and hinder the ubiquitination of IRF3 (Shi et al., 2010). Therefore, IRF3 can maintain its own activity after the modification of ISG and improve the IRF3-mediated antiviral response by inhibiting its own degradation.

ISG15 can bind to protein kinase R (PKR), an IFN-inducible protein kinase activated by double-stranded RNA. Simultaneously ISG15 can also activate PKR in the absence of viral RNA. Activated PKR can inhibit protein translation by phosphorylation of eukaryotic initiation factor 2α, and PKR activated by ISG15 can further promote IFN production (Okumura et al., 2013). In addition, RIG-I is the target protein of ISG15, and RIG-I can activate the RNA sensors of IRF3 and NF-κB. The covalent combination of ISG15 and RIG-I can downregulate signal transduction mediated by RIG-I. Free ISG15 can regulate the level of RIG-I by promoting the interaction between RIG-I and the autophagy substrate protein p62 (Nakashima et al., 2015; Du et al., 2018). ISGylation of phosphorylated STAT1 can also maintain its activity by inhibiting its own polyubiquitination and proteasomal degradation (Ganesan et al., 2016). In another example, ISGylation of filamin B can negatively regulate IFN-α-mediated c-Jun N-terminal kinase signals and inhibit cell apoptosis (Jeon et al., 2009). ISG15 can also bind to ubiquitin-conjugating enzyme 13 to inhibit the ubiquitination of transforming growth factor kinase one and negatively regulates the NF-κB pathway (Takeuchi and Yokosawa, 2005).

**FIGURE 3:**
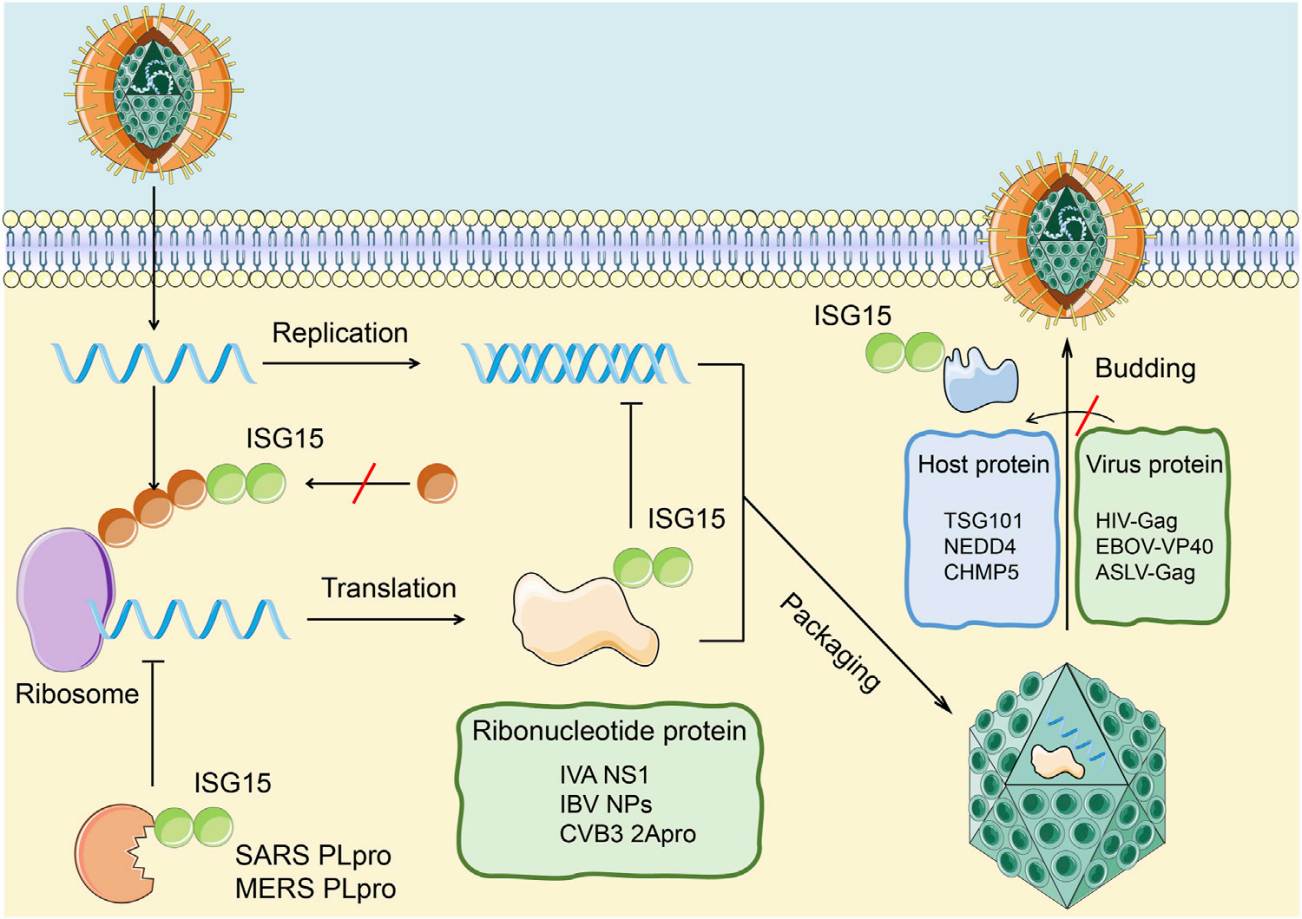
Antiviral effects of ISGylation on host and viral proteins. ISG15 affects the infection of cells by the virus through covalently binding with viral proteins and host proteins. 1. The combination of ISG15 and the viral nucleoprotein (green) can destroy the protein oligomerization and the ability of the viral nucleoprotein to inhibit virus replication. The ubiquitin-like modification formed by this combination can be cleaved by PLpro to restore the replication ability of the virus.2. The combination of ISG15 and host protein (blue) can inhibit the interaction between host protein and virus protein, thereby inhibiting the release of virus particles in the cell.

On the one hand, ISG15 influences antiviral immunity by ISGylation of host cell proteins and the relevant immune signaling pathways. On the other hand, ISG15 can affect virus replication, release, and latency in the host body through the ubiquitin-like modification of the virus protein to achieve antiviral immunity (Figure 3). Relevant examples are described in detail below.

## ISGylation of Viral Proteins and Their Functions

Lenschow and Werneke’s team demonstrated that ISG15-knockout mice were more susceptible to IAV and IBV, herpes simplex virus, norovirus, chikungunya virus, and other pathogens than wild-type mice (Lenschow et al., 2007; Werneke et al., 2011; Morales and Lenschow, 2013; Rodriguez et al., 2014). They demonstrated that both free and binding ISG15 expression is upregulated after pathogen infection, and both forms of ISG15 exhibit antiviral activity. For example, after IAV infection, free ISG15 can bind to the NS1 protein with seven lysine residues, which are potential target sites for ISGylation, blocking the nuclear localization of the NS1 protein and inhibiting virus replication, RNA processing (Jumat et al., 2016). At the same time, the ISGylation of NS1 can inhibit the interaction with PKR, which relieve the inhibition of NS1 protein on innate immunity and restoring IFN-induced anti-IAV activity (Pincetic et al., 2010).

As for IBV, nucleoprotein and matrix protein M1 are also targets for covalent binding of ISG15. Nucleoprotein ISGylation hinders the oligomerization of a large number of other non-conjugated nucleoproteins, inhibits the formation of IBV ribonucleic acid protein, and reduces viral protein synthesis and viral replication (Durfee et al., 2010; Zhao et al., 2016). Rahnefeld et al. found that the coxsackie virus CVB3 2A protease ISGylation can inhibit the cleavage of eukaryotic translation initiation factor 4 G and reduce CVB3 replication (Rahnefeld et al., 2014).

Studies have shown that ISG15 can affect the release of HIV, Ebola virus, and avian sarcoma leukosis virus through different mechanisms. Pincetic and Okumura demonstrated that ISG15 inhibited the monoubiquitination of the HIV group-specific antigen protein, blocked its interaction with host tumor susceptibility gene 101, and inhibited the emergence and release of HIV (Okumura et al., 2006). When infected with Ebola, the ubiquitin ligase NEDD4 catalyzes the ubiquitination of the viral matrix protein VP40 and promotes the release of virus-like particles (Okumura et al., 2008). Lenschow and Malakhova demonstrated that ISG15 inhibits the transfer of ubiquitin-binding enzyme to NEDD4 and activity of NEDD4 ubiquitin-binding enzyme, thus inhibiting the budding and release of the Ebola virus (Malakhova and Zhang, 2008). ISGylation of charged multivesicular body protein 5 (CHMP5), a component of the endosome sorting complex, promotes its aggregation and the isolation of Vps4 coenzyme factor LIP5 and limits the membrane recruitment of Vps4 and its interaction with the avian sarcoma leukosis virus budding complex, thereby inhibiting the release of intracellular virus-like particles (Pincetic et al., 2010). In addition, researchers found that ISG15 can also affect the budding process of vesicular stomatitis virus by inhibiting the activity of NEDD4 and that ISG15 overexpression can significantly reduce the viral titer of its wild-type strains (Malakhova and Zhang, 2008).

Another study showed that ISG15 regulated the incubation period of the virus. Dai et al. used Illumina microarray technology to analyze the gene expression changes in primary human oral fibroblasts after infection with Kaposi’s sarcoma-associated herpes virus and found that a series of IFN-stimulated genes were upregulated, especially ISG15 and ISG20, which maintain the virus incubation period by regulating Kaposi’s sarcoma-associated herpes virus-specific microRNA (Dai et al., 2016). This reduces the expression of ISG15 during the incubation period of Kaposi’s sarcoma-associated herpes virus infection and increases the expression of virus cleavage genes and the release of virus particles.

These results suggest that free or bound ISG15 produced by stress can regulate the function of viral proteins, inhibit viral replication, budding, and release. Thus, ISG15 may play a key role in inhibiting viral infection (Table 1).

**TABLE1:** Interaction between ISG15 and viral proteins.

Viral proteins	Biological effects after ISGylation	Impact on viral infection	Reference
IVA NS1	ISG15 inhibits viral proteins nuclear translocation and restores host antiviral responses	Inhibits IBV replication	(Tang et al., 2010; Zhao et al., 2010)
IBV NPs	ISGylation of NPs inhibit the oligomerization of unmodified NPs, which impedes viral RNA synthesis	Inhibits IBV replication	Zhao et al. (2016)
CVB3 2Apro	ISG15 inhibits its protease activity to restore host protein translation	Inhibits CVB3 replication	Rahnefeld et al. (2014)
HIV Gag	ISG15 inhibits the monoubiquitination of Gag protein and block its interaction with TSG101	Inhibits the emergence and release of HIV.	Okumura et al. (2006)
EBOV VP40	ISGylation of NEDD4 ubiquitin-binding enzyme inhibits its interaction with VP40	Inhibits the budding and release of Ebola virus	(Yasuda et al., 2003; Okumura et al., 2008)
ASLV Gag	The ISGylation of CHMP5 limits the membrane recruitment of Vps4 and its interaction with the ASLV Gag	Inhibits the ASLV budding complex, then inhibits the release of intracellular virus-like particles	Pincetic et al. (2010)
SARS PLpro MERS PLpro	PLpro protease is a virus-encoded DUB, which active on ubiquitin like molecule ISG15	Negatively regulates the innate immune response to the virus	Rut et al. (2020)

## ISG15 Participates in Non-Viral Innate Immune Responses

Recent work has also highlighted the function of ISG15 in non-viral innate immune responses, such as pathogen defense responses, host damage and repair responses, and other host signaling pathways. ISG15^−/−^ mice are more susceptible to *mycobacterium* than wild-type mice, verifying that the degree of *mycobacterium* drop is not a determinant of susceptibility enhancement. Significantly increased cytokine release was detected in ISG15^−/−^ mice, and the cytokine storm induced by ISG15 knockout was blocked by tumor necrosis factor-α-specific antibodies (Bogunovic et al., 2012; Kimmey et al., 2017). During *Listeria monocytogenes* infection, the expression of ISG15 increases, which depends on the cytosolic DNA-sensing pathway, and enhanced secretion of IL-6 and IL-8 was detected in ISG15-overexpressing cells (Radoshevich et al., 2015). These studies demonstrate that ISG15 plays an antagonistic role in the host response to pathogens and regulates cytokine signal transduction. Exogenous stimuli, such as DNA damage, radiation, ischemia, and telomere shortening, can also induce immune cells to produce ISG15 (Liu et al., 2004).

## SARS-CoV-2 Papain-Like Protease: A Deconjugating Protease

The SARS-CoV–coronavirus genome encodes two viral proteases: PLpro and 3C-like protease. The structure and function of PLpro has been a hot topic in the molecular biology of coronavirus recently. PLpro is involved in cutting the N-terminal part of the SARS-CoV replicase polymerin and is a regulatory protein molecule for the formation of the SARS-CoV replicase complex (Shin et al., 2020). Results showed that SARS-CoV PLpro protease is a virus-encoded DUB, which has an obvious deubiquitinating effect on cellular proteins (Klemm et al., 2020). PLpro is also active against ubiquitin and ISG15, which can negatively regulate the innate immune response to the virus (Shin et al., 2020). There are also OTU domain-containing proteases that can be encoded by Crimean-Congo hemorrhagic fever orthonairovirus, porcine reproductive and respiratory syndrome virus, and equine arteritis virus, which have properties similar to those of PLpro (Frias-Staheli et al., 2007). These proteins have been shown to reduce ubiquitin and ISG15 conjugates in cells. However, the researchers have compared SARS-CoV-2-PLpro with similar enzymes of other coronaviruses (SARS-CoV-1 and MERS). It was found that the SARS-CoV-2-PLpro enzyme processes ubiquitin and ISG15 in a different way with SARS-CoV-1-PLpro (Rut et al., 2020).

Recently, Huang and Zhang have made progress in elucidating the complex structure of SARS-CoV-2 PLpro and antiviral drug discovery (Fu et al., 2021). They found that the small molecule inhibitor GRL0617 inhibited the activity of PLpro to shear the ubiquitin-like chain and the ubiquitin-like protein ISG15 chain *in vitro* and the ability to inhibit viral replication of SARS-CoV-2. The structure of the inhibitor and protein complex and two-dimensional NMR experiments revealed that GRL0617 interferes with protein–protein interaction between PLpro and ISG15, acting as an inhibitor for this interaction. They established that SARS-CoV-2 protease PLpro is a target for antiviral drug development at the cellular and atomic resolution crystal structure levels and identified the binding site of GRL0617 as a hot spot for antiviral drug development targeting PLpro using a variety of biophysical methods.

## Discussion

ISG15 is a ubiquitin-like protein, produced by IFN, viruses, lipopolysaccharides, and other stimuli. ISG15 exerts antiviral effects by covalently binding to target proteins, inhibiting the release and replication of viral particles, and regulating the incubation period of viruses. In addition to the ISG15 covalent conjugate, the ISG15 monomer can promote the proliferation of NK cells and dendritic cells and enhance the chemotactic activity of neutrophils. Moreover, ISG15 is implicated in host damage, DNA repair, autophagy, protein translation, and other processes. ISG15 is also associated with the occurrence of cancer. However, there are still many unsolved mysteries about the biological function of ISG15 and the molecular mechanism underlying the antiviral effects of the ubiquitin-like modification system.

The PLP2 domains of many human and animal coronaviruses, such as the SARS coronavirus, MHV-A59, NL-63, and 229E, have demonstrated DUB activity, and the catalytic sequence of the PLP domain of these coronaviruses is highly conserved. However, it is still unclear whether DUB activity and regulation of the host natural immune response are the common characteristics of all PLpros, and the functional relationship between DUB activity of PLpro and its IFN antagonism needs further study.

In general, the diversity and broad spectrum of substrates, complexity of the ISG enzyme system, and cross-linking with the ubiquitination pathway all determine the complexity of ISG15 function. Further understanding of the molecular trajectory of the ubiquitin-like protein ISG15 may lead to new therapeutic strategies for antiviral treatment, immune function regulation, and cancer treatment.
